# Automated generation of Kochen-Specker sets

**DOI:** 10.1038/s41598-019-43009-9

**Published:** 2019-05-01

**Authors:** Mladen Pavičić, Mordecai Waegell, Norman D. Megill, P. K. Aravind

**Affiliations:** 1 Department of Physics—Nanooptics, Faculty of Math. and Natural Sci. I, Humboldt University of Berlin, Berlin, Germany; 2 Center of Excellence CEMS, Photonics and Quantum Optics Unit, Ruder Bošković Institute, Zagreb, Croatia; 3 Institute for Quantum Studies, Chapman University, Orange, CA 92866 USA; 4 Boston Information Group, Lexington, MA 02420 USA; 5 Physics Department, Worcester Polytechnic Institute, Worcester, MA 01609 USA

**Keywords:** Quantum information, Theoretical physics

## Abstract

Quantum contextuality turns out to be a necessary resource for universal quantum computation and also has applications in quantum communication. Thus it becomes important to generate contextual sets of arbitrary structure and complexity to enable a variety of implementations. In recent years, such generation has been done for contextual sets known as Kochen-Specker sets. Up to now, two approaches have been used for massive generation of non-isomorphic Kochen-Specker sets: exhaustive generation up to a given size and downward generation from master sets and their associated coordinatizations. Master sets were obtained earlier from serendipitous or intuitive connections with polytopes or Pauli operators, and more recently from arbitrary vector components using an algorithm that generates orthogonal vector groupings from them. However, both upward and downward generation face an inherent exponential complexity barrier. In contrast, in this paper we present methods and algorithms that we apply to downward generation that can overcome the exponential barrier in many cases of interest. These involve tailoring and manipulating Kochen-Specker master sets obtained from a small number of simple vector components, filtered by the features of the sets we aim to obtain. Some of the classes of Kochen-Specker sets we generate contain all previously known ones, and others are completely novel. We provide examples of both kinds in 4- and 6-dim Hilbert spaces. We also give a brief introduction for a wider audience and a novice reader.

## Introduction

Quantum contextuality arguably plays an important role in the field of quantum communication and quantum computation. In this paper we shall focus on the most explored and used contextuality configurations–the so-called Kochen-Specker (KS) sets. We begin with a review of some important concepts.

Imagine a system of nonlinear equations describing a relation between vectors or states. Let the vectors be 3-dim (3D) ones, forming triplets: ***a***_*l*_ = *a*_*lx*_**i** + *a*_*ly*_**j** + *a*_*lz*_**k**, *l* = 1, 2, 3, and let the relation be the orthogonality ***a***_*l*_ · ***a***_*m*_ = 0, *l*, *m* = 1, 2, 3, *l* ≠ *m*. Let us have many such triplets interconnected within a system, and let us ask ourselves whether we can assign 0 and 1 to them in such a way that just one vector in any triplet is assigned 1. Now we have two problems for a chosen system. One is the assignment problem of finding a 0–1 distribution (or proving its impossibility), and the other is the coordinatization problem of finding vector components. Both are of exponential complexity. All the supercomputers on Earth would take an “age of the universe” amount of CPU time to solve either of the two problems, even for some of the simplest systems. Now imagine three dots (we call them *vertices*) on a paper or a screen connected by a curve (we call it an *edge*). Connect them so as to follow the pattern in which the aforementioned vector triplets are connected within their system. They will form a so-called *hypergraph* and it has been recognized that the two representations are equivalent: vertices correspond to vectors, and edges to orthogonalities. And finding solutions of the assignment problem for hypergraphs statistically proves to be a feasible task on supercomputers and clusters, typically reducing “age of the universe” run times to anywhere from CPU seconds to a few CPU weeks depending on the size and nature of the hypergraphs of interest. A hypergraph with a solution is a classical noncontextual system, and the one without it is a quantum contextual KS set. The coordinatization problem was tougher. Finding vector components that can be assigned to vertices was still a task of exponential complexity. A number of workarounds have been designed, until most recently we turned the problem upside-down. Instead of searching for vector components we might assign to chosen hypergraphs, we search for hypergraphs we might assign to chosen vector components, the so-called *master sets*. But even that does not enable a satisfactory efficent generation of KS sets and to achieve this goal, in the present paper, we elaborate on features, algorithms, and methods which not only speed up the search for KS sets almost exponentially but also enable (reasonably) arbitrary exhaustive generation of KS sets from master sets. The details and references are given below.

A series of experimental implementations of 4D KS sets have been carried out recently, using photons^1–6^, neutrons^7–9^, trapped ions^10^, and solid state molecular nuclear spins^11^. Sets in 6D have been implemented via six paths^12,13^ and in 8D using photons^14^.

These experiments have been implemented either under assumption of predetermined noncontextual values or under the *faithful measurement condition*^15,16^ meaning that they did not overcome the noise-induced finite-precision loophole. It is, however, possible to formalize contextuality in a noise-robust manner and develop experimental tests for them^17–20^. It is also possible to modify the Kochen-Specker notion by appealing to *ontological faithfulness*^21^.

Our theoretical interest in contextual KS sets is justified by the following recent achievements.

A connection between contextuality and universal quantum computation has been established to an extent that makes contextuality a necessary resource for universal quantum computation. A pressing open question for qubits is whether a suitable operationally motivated refinement or quantification of contextuality can provide a quantum speed-up^22^. Also, one can design quantum gates via operator-generated 4D complex KS sets^23^.

In quantum communication, KS sets might protect^24^ and secure^25^ quantum key distribution (QKD) protocols.

In lattice theory, KS sets have served as generators of higher-order generalized orthoarguesian lattices^26,27^.

For the above applications of KS sets, especially in quantum computation, it is important to generate sufficiently large sets to enable varieties of different implementations and to characterize their main features and information on their structure.

To date, all papers on KS sets in the literature have been focused on

(i) discovering or implementing smallest sets (e.g.^28^), or on
(ii) an exhaustive upward hypergraph-generation of sets (see the introductory paragraph of Results), e.g.^29^, or on
(iii) a top-down, random downward generation of sets (see the introductory paragraph of Results) from fortuitously obtained master sets (e.g.^23,30–39^), or on
(iv) generating sets in higher dimensions from the ones in smaller dimensions (e.g.^40–42^), or on
(v) generating big master sets from simple vector components in order to compare them with master sets obtained via (i–iii) methods^43^.

Advantages and disadvantages of these approaches are as follows.

(i) The smallest KS sets are of just historical relevance, because practically all of them in even dimensional (4D through 32D) Hilbert spaces are already known. Besides, all of them are but by-products of our current generation algorithms;
(ii) At least for the time being, exhaustive upward hypergraph-generation of KS sets faces computational limits of supercomputers and is limited to ca. 40 hypergraph vertices in 3D, to ca. 25 ones in 4D, and so on. Still, this kind of a generation remains the only deterministic and exhaustive one.
(iii) Big master sets which serve for random downward generation of smaller sets are based on serendipitous or intuitively found connections of KS hypergraphs with polytopes or Pauli operators which we run out of and which do not generalize.
(iv) No universal constructive algorithm for arbitrary input KS sets from smaller dimensions has been proposed for a potential automated generation of sets in higher dimensions. However, the approach might be the best option for big sets and dimensions where other methods face computational limits.
(v) The algorithms and programs are computationally feasible for generating master sets from the vector components that are already known to yield KS masters obtained via other methods (i–iii), even on a PC (this is what has been carried out in ref.^43^). However, they are not computationally feasible, even on supercomputers, for random computer generated components without additional analyses, algorithms, and programs we provide in the present paper. For instance, in ref.^43^ (Table 2) we were not able to obtain smaller KS sets from the 4D 2316–3052, or 6D 11808–314446, or 8D 3280–1361376, or 16D, or 32D master sets directly. The main reason is that we cannot generate smaller sets from a master set by a brute force–the programs are too slow for that. E.g., in 6D, without a new algorithm for splitting the original KS master 834–1609 (obtained from the vector components in ref.^43^) into two smaller sub-masters (as we do here), we would not have been able to arrive at the distribution given in Fig. 5 and results in Fig. 4(e–g). Taken together, the main aim of ref.^43^ was to classify KS sets in even dimensions via vector-component generated master sets that would contain all known KS sets, while the main aim of the present paper is to provide a functional and feasible method of generating KS sets of chosen sizes and properties from such master sets.

In the present paper, we present an optimized method of generating KS sets from tweaked KS master sets engineered via the method outlined in ref.^43^. In the latter method, we start with random vector components and build *n*-tuples of mutually orthogonal vectors which results a in KS masters with a specific coordinatization. In the former method, the components are filtered by features of KS sets obtained from coordinatizations of already known KS sets as exemplified below (see figures in the main body of the paper and in the Supplementary Material). This provides us not only with a uniform and general method of computationally feasible KS set generation, but also with a larger scope and a bigger, more thorough, picture of quantum contextuality than any of the previous approaches. That comes at a price of engaging supercomputers to arrive at results, but once obtained, they can be verified either by hand or via a graphical representation as given by our figures.

Experimentally, the complexity of KS set implementation grows only linearly with the complexity of their structure and, on the other hand, the simplest KS sets often do not possess features that bigger KS sets exhibit, such as the so-called *δ*-feature^39,43^ (see Fig. 3 and defined in MMP Hypergraph Language and Vector Generation of KS Sets from Basic Vector Components below), absence of coordinatizations with real vectors, and the absence of parity proofs.

Since the proposed approach charts a new territory, we limit ourselves to 4D space and an example from 6D space, but we stress that the approach can be applied to any dimension. Also, we answer several recently posed open questions.

In this paper, to achieve automated generation of KS sets we rely on our hypergraph language and its algorithms and C programs first given in^29^ and extended here, analogous to the recently developed algorithm and program Melvin^44^ whose authors carried out an “Automated Search for New Quantum Experiments.” We share their guiding principle: “In contrast to human designers of experiments, [*our computer programs*] do not follow intuitive reasoning about the physical system and, therefore, lead to the utilisation of many unfamiliar and unconventional techniques that are challenging to understand”^44^.

## Results

**Figure 1:**
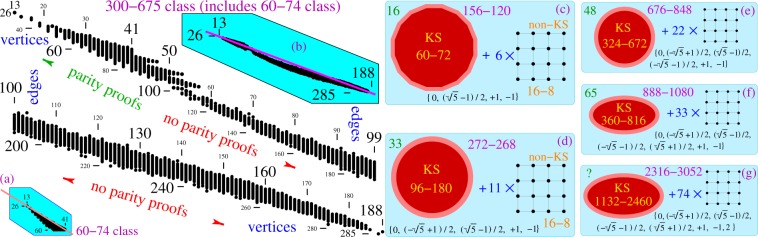
Distribution of 10^10^ non-isomorphic critical KS sets from the 300–675 class we found by means of programs Mmpstrip, States01, and Mmpsubgraph. Dots represent KS criticals with the number of edges given at the ordinates and the number of vertices at the abscissae. When dots are next to each other (“glued” together), they are shown as vertical lines; e.g., at the far right end of the upper strip, there is a line of KS criticals with 99 edges ranging from 168 to 183 vertices, then, just below it, there is a 184–99 gap, followed by a dot which represent KS criticals with 99 edges and 185 vertices. Vertex units are scaled to 1/4 of edge units for a compact presentation. Inset (**b**) gives the whole span of criticals and shows that the criticals are agglomerated below the line which connects the smallest and largest criticals. Insets (**c**–**g**) show vector generated sets with the given coordinatization: KS hypergraphs for each of them are represented via biggest outer polygon loops found by Loop (after searching through 50,000 combinations for each): 16 edges (**c**), 33 (**d**), 48 (**e**), 65 (**f**), and ‘?’ (**g**), indicating that we did not run Loop on them because it would take too much CPU time–cf. hexagon 18–9 in Fig. 1 of the Supplementary Material.

The main result of the paper is that via a rather simple algorithm we can generate any class of KS sets from basic real or complex vectors. Let us consider the following example. In the last 27 years several research groups (Kochen, Specker, Peres, Cabello, Pavičić, Megill, McKay, Merlet, Aravind, Waegell, and others–see Fig. 1 and Sec. III in^39^) obtained 1233 4D KS sets by investing a considerable amount of research work and CPU-centuries of calculation on supercomputers which they published in over 20 papers. In contrast, with the help of our algorithm, by just putting the three components of their vectors {−1, 0, 1} in its program, we obtain all those KS sets and additional ones containing them, in seconds on a PC.

So what is a KS set? It is a set of *n*-tuples of mutually orthogonal vectors that provides a constructive proof of the Kochen-Specker theorem. This theorem states that it is impossible to ascribe predetermined eigenvalues to all quantum observables, which we call the contextuality of quantum mechanics. We represent the *n*-tuples as edges on a hypergraph, with individual vectors assigned to the vertices of the hypergraph.

*Kochen-Specker theorem*^40,45^ reads: In ${ {\mathcal H} }^{n}$, *n* > 3, there are sets of *n*-tuples of mutually orthogonal vectors to which it is impossible to assign 1 s and 0 s in such a way that (i) No two orthogonal vectors are both assigned the value 1 and (ii) In any group of *n* mutually orthogonal vectors, not all of the vectors are assigned the value 0.

These sets are called *KS sets* and the vectors *KS vectors*.

That means that the terms “generation of hypergraphs” and “downward generation” used in the text are tantamount to the term “generation of KS sets” used in any of the papers mentioned in points (i–v) in the Introduction and to the term “generation of KS sets from master sets” used in (iii) and (v), respectively.

Generating hypergraphs and checking them for the KS property are exponentially complex tasks in general but for the great majority of runs they turn out to run in a feasible amount of time due to various heuristics and other techniques incorporated into our algorithms. So, for instance, our programs can generate and assign vectors to hypergraphs with over 100,000 vertices and edges. Verification of the KS property becomes very demanding on supercomputers when the number of vectors/vertices exceeds 1,000 and the number of dimensions exceeds 10, though.

We limit ourselves to critical non-isomorphic KS sets, where *critical* means that they are minimal in the sense that removing any *n*-tuple of mutually orthogonal vectors (i.e. any hypergraph edge) turns a KS set into a non-KS set. In other words, they represent non-redundant blueprints for their implementation since bigger KS sets that contain them only add orthogonalities that do not change the KS property of the critical sets. Note that edges within a hypergraph correspond to measurements necessary to show contextuality.

To deal with hypergraphs conveniently in our computer programs, we make use of the McKay-Megill-Pavičić (MMP) hypergraph language^29,39^.

### MMP hypergraph language

We describe KS sets by means of McKay-Megill-Pavičić (MMP) hypergraphs, which are defined as hypergraphs in which edges contain at least *n* vertices (corresponding to orthogonal *n*-tuples of vectors) and intersect each other in at most *n* − 2 vertices (corresponding to vectors themselves). MMP hypergraphs are encoded by means of printable ASCII characters^29^. Vertices are denoted by one of the following characters: 1 2 …9 A B …Z a b …z ! “ # $ % &’ () * - / : ; < = > ? @ [ \ ] ˆ _ ‘ { | } ∼^29^. When all of them are exhausted we reuse them prefixed by ‘+’, then again by ‘++’, and so forth. An *n*-dim KS set with *k* vectors and *m n*-tuples is represented by an MMP hypergraph with *k* vertices an *m* edges which we denote as a *k* − *m* set. A KS set with a parity proof is one with an odd number of edges, in which all vertices belong to an even number of those edges, which makes the impossibility of a 0–1 assignment transparent.

A number of examples are given below. We generate, process, and handle MMP hypergraphs by means of algorithms in the programs Shortd, Mmpstrip, Mmpsubgraph, Vecfind, States01, and others. See the Supplementary Material for more details.

Note that the aforementioned *n* − 2-intersection condition is the only condition that restricts a general hypergraph to an MMP hypergraph^29^. It also directly generates the *δ*-feature (two edges might share *n* − 2 vertices) we introduce in Vector Generation of KS Sets from Basic Vector Components below.

### Coordinatization of KS Sets

As we mentioned at the beginning of this section, we can generate the aforementioned KS sets from nothing but three vector components {−1, 0, 1} provided to program Vecfind.

It works as follows. We specify a dimension *n* (4 in this example), a set of vector component values {0, ±1}, and the option master. (In general, the vector component values can be arithmetic expressions involving complex numbers, including exponential and trigonometric functions). The program builds an internal list of all possible non-zero vectors containing these components. (The vectors are not normalized in general. If two vectors are proportional, one of them is discarded). From this list, it finds all possible mutually orthogonal *n*-tuples of vectors. It then generates a hypergraph (in the MMP notation) with edges corresponding to these mutually orthogonal *n*-tuples. We call this hypergraph a “master set.” Depending on the vector component values chosen, this master set may or may not have the KS property. The program States01 tests for this property, and master sets without the KS property are discarded. When that happens, we can add more vector component values or try a different set of vector component values. Once we obtain a master set with the KS property, we can use our already existing programs (developed for our previous work with master sets obtained from polytopes, etc.) to extract critical KS subsets. Although in one sense the master option algorithm is straightforward–it just exhausts all possible mutually orthogonal *n*-tuples that can be built from given vector components–it nonetheless creates master sets that contain all the master sets of our previous work derived from polytopes or Pauli operators, when we provide it with nothing but the same vector components.

In this example, the set we obtain is indeed a KS master set with 40 vertices and 32 edges (See Fig. 1 in Supplementary Material). It reveals that Peres’ 24-24 set obtained previously is not a *proper* master set in the sense of generating all KS subsets with the coordinatization (assignment of vectors) generated by the vector components of the Peres coordinatization. Details are given in the Supplementary Material.

### Polytope vs. Vector generation

In the last ten years, various KS sets have been generated from a number of polytopes for which correspondences with KS hypergraphs have been found^23,30–38^. These correspondences have proven very useful in the development of the more systematic approach to the generation of KS sets developed in this paper.

The vertices of regular or highly symmetrical polytopes in a number of dimensions, in both real and complex spaces, can be mapped into numerous KS master sets in Hilbert spaces. In particular, the 24-cell, 600-cell and 120-cell, which are the three exceptional 4D regular polytopes (having no analogs in lower or higher dimensions) provide striking illustrations of this statement. All these polytopes have the feature that their vertices lie on the surfaces of 3D-spheres and come in antipodal pairs whose members are diametrically opposite each other on the 3D-sphere. Since each antipodal pair maps on to a single ray, each of these polytopes gives rise to half as many rays as its vertices. The 24-, 600- and 120-cells thus lead to 12, 60 and 300 rays, respectively.

Furthermore, the vertices of each of these polytopes contain many inscribed copies of the cross-polytope (or 16-cell), each of which give rise to 4 mutually orthogonal rays, and thus corresponds to an edge in an MMP diagram. Naturally, only polytopes which give rise to edges can give rise to KS sets, and so, not all polytopes have a direct mapping to a KS master, nor do all KS masters have a recognized mapping from a polytope.

In the case of the 24-cell, one must unite the 12 rays with a second set of 12 rays obtained from its dual (another 24-cell, but oriented differently from the first) to obtain a set of 24 vertices and 24 edges that is identical to the famous 24–24 set in 4-dimensions discovered originally by Peres and then explored by a number of other authors.

The 600-cell gives rise to a 4D KS master set of 60 vertices that form 75 edges of mutually orthogonal vertices. By stripping only one edge at the time from this set using MMPSTRIP, we obtain 75 isomorphic 60–74 KS subsets which merge into a single 60–74 set. Their subsets can therefore be called the 60–75 and 60–74 classes, respectively. They are identical, short of the 60–75 set from the 60–75 class^38^.

The 120-cell, which is the dual of the 600-cell, is unusual in that it contains ten 600-cells as subsets within it. Further, these ten 600-cells divide into two sets of five, with the members of each set having no vertices in common and the two together covering all the vertices of the 120-cell. From this it follows that the corresponding quantum 300–675 KS set has ten different 60–75 sets within it as subsets.

It should be noted that the members of the 60–74(75) class have vector components from the set ${\mathscr{V}}=\{0,\pm \,(\sqrt{5}-1)/2,\pm \,1,\pm \,(\sqrt{5}+1)/2,2\}$^33^, while those of the 300–675 class have vector components derived from the elements of matrices based on ${\mathscr{V}}$^36^.

This might suggest that the vector sets generated from the aforementioned coordinates would lead to not just the 24–24, 60–74(75) and 300–675 classes, but also the inclusion of the second of these within the third. However this turns out not to be the case at all. The set ${\mathscr{V}}$ unexpectedly generates a KS master set, 2316–3052, consisting of 2316 vertices and 3052 edges, whose class of KS sets is too large to be determined by the present methods. But what is even more interesting is that we need not employ all the components of ${\mathscr{V}}$ to get the members of the 60–74 class. Removing just 2 from ${\mathscr{V}}$ gives us a 888–1080 master set, the further removal of $(\sqrt{5}+1)$/2 gives a 676–848 master set, the still further removal of $(\,-\,\sqrt{5}-1)$/2 → a 272–268 master set and, finally, the removal of $(\,-\,\sqrt{5}+1)$/2 → a 156–120 master set. In other words, we have the nesting of master sets described by the relation 156–120 ⊂ 272–268 ⊂ 676–848 ⊂ 888–1080 ⊂ 2316–3052, with the largest of these having all elements of ${\mathscr{V}}$ as coordinates of its vectors and each member of the sequence having one less element than the member above it.

As was the case with the 40–32 master set discussed in the previous subsection, each of the five master sets just described can be decomposed into two disconnected pieces. The smallest of them, 156–120, consists of a 60–72 KS set and 6 structurally identical non-KS 16–8 sets, which is conveniently expressed by the equation 156 − 120 = 60 − 72 + 6 × 16 − 8. In an exactly similar way, the decompositions of the other master sets can be described by the equations given in Fig. 1. All 16–8 sets have the same hypergraph representation as the one shown in the right part of Fig. 1 of the Supplementary Material, i.e., they are all isomorphic to each other.

The above master sets might well contain the 60–75 and 300–675 classes, but we did not pursue this point (although we did verify that they contained a large number of KS sets from these classes). We demonstrated earlier that the 300–675 class contained both the 60–74(75) and 96–96 classes^35^, and filled in the gap between the 82–41 and 211–827 sets we were left with in^38^. This allowed us to assemble all the information we have presented in Fig. 1 and in the Supplementary Material.

In Fig. 1 we see that the 60–74 class exhibits a *cut-off* feature from below which we find, via both the polytope and vector generation, to be an indication of the existence of a bigger set which contains it. There are actually several such cut-offs in the figure which form saw-tooths at the levels of 60, 72, 84, and 96 vertices which might correspond to the consecutive vector generated sets above. There is also a spindle-shaped agglomeration of a more complete distribution of sets as shown in Fig. 1. These features are elaborated in detail in the Supplementary Material, and they are our starting points for a pure vector generation of supersets in the next subsection.

### Vector generation of KS Sets from basic vector components

The theoretical background and algorithms of quantum computation are to a large extent based on qubits and their states, including entanglements in particular. KS sets should therefore also allow a qubit state representation. This can be done so as to define them via operators whose eigenvectors (eigenstates) then directly define vertices of MMP hypergraphs in the complex 4D ${ {\mathcal H} }^{2}\otimes { {\mathcal H} }^{2}$ Hilbert space. Via that approach we shall get the coordinatization for our vector generation.

We carried out the qubit approach in^23^ by means of Pauli operators for two qubits and their eigenstates and generated the 60–105 KS class. In^23^ we obtained KS sets with an odd number of edges with the help of parity proof programs, and in^39^ we generated sets with even numbers of edges and also all those ones with an odd number of edges which do not have parity proofs via our MMPSTRIP and STATES01 programs. The master set in MMP hypergraph notation is given in [^39^, App. 2, p. 22]. A number of 60–105 KS class figures and MMP hypergraphs are given in^39^.

The (eigen)vectors we obtained have components in the complex field, while Peres’ 24–24 set and the whole 24–24 class is properly contained in the 60–105 class, which originally used real coordinatization. Bigger sets from the 60–105 class do not allow simple real coordinatization, e.g., with components from {−1, 0, 1} as we have shown in the Supplementary Material, but they might allow an intricate kind of real coordinatization which are not eigenvectors of the Pauli operators that we used to generate the 60–105 KS class. So, such real vectors might allow a representation by means of particles with four level spin $s=\frac{3}{2}$ (verifiable via, e.g., a Stern-Gerlach device), so that dim${ {\mathcal H} }_{s}$ = 2*s* + 4 is satisfied, but possibly not via two qubits (dim (${ {\mathcal H} }^{2}\otimes { {\mathcal H} }^{2}$) = 2^2^ = 4). This prompted us to investigate to what extent KS sets are determined by coordinatizations that they do or do not allow, and whether a coordinatization itself can enable us to generate bigger sets and master sets.

**Figure 2:**
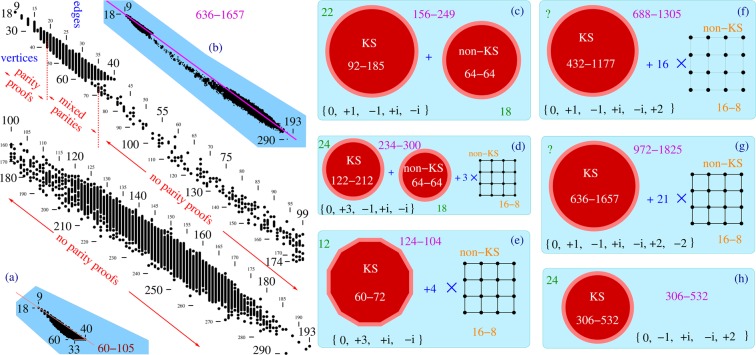
Distribution of KS non-isomorphic criticals from the 636–1657 class. 7,720,530 criticals from the 60–105 subclass are taken over from Fig. 4 of ^39^ and 12,952 higher criticals are generated for this paper. For the representation of the criticals via dots and lines in the main figure, see the caption of Fig. 1. The 60–105 KS criticals exhibit a flat cutoff feature at the level of 60 vertices, inset (**a**), similar to the one shown in the inset (**a**) of Fig. 1; The global distribution is shown in the inset (**b**); smaller master sets generated from smaller sets of vector components and mostly consisting of a KS set and several isomorphic non-KS sets are shown in insets (**c**–**h**); (**f**,**g**) ‘?’ again means that it would take too much CPU time to run Loop on the sets.

In^23,39^ we presented in detail how the operator structure and related eigenstates (eigenvectors, vertices) were obtained from the 600-cell polytope and how the whole 60–105 class was generated and which features its KS sets possess. The eigenvectors had the components from the set ${\mathcal I}$ = {0, ±1, ±*i*}. However, the class also exhibits a flat cutoff at the 60 vertex level of critical KS sets shown in Fig. 2(a), similar to the one of the 60–74 class, shown in Fig. 1(a). That indicated that ${\mathcal I}$ might generate a much bigger master set containing the 60–105 one, although, to our knowledge, no one has come forward with such a set. We are now confirming the conjecture by means of a direct vector generation from ${\mathcal I}$.

So, we started our vector generation with components from ${\mathcal I}$ and then pursued its variations via an automated computer search. From ${\mathcal I}$ we arrive at the 156–249 master set whose KS subset 92–185 is already much bigger than the 60–105 that is contained in it. By stripping down its edges via MMPSTRIP and filtering with STATES01, we form the 92–185 class. By adding 2 to ${\mathcal I}$ ({0, ±1, ±*i*, 2}), we get 688–1305, and by adding −2 to it ({0, ±1, ±*i*, ±2}) we obtain the 972–1852 master and the class 636–1657 which contains all the others. They are shown in Fig. 2(c,f,g). As the master sets from the previous section, they also consist of smaller KS and non-KS sets. However, the non-KS sets are not always 16–8 ones but also 64–64 ones as in Fig. 2(c,d). All 16–8 sets are isomorphic to each other, thus effectively reducing all the masters to two sets: one KS and one non-KS. Sometimes we do not get a non-KS set at all, as e,g., in Fig. 2(h) when all the vectors are exhausted in building the KS set. We were unable to find correlations between kinds of non-KS set and vector components indicated at the bottom of Fig. 2(c–h).

Each set of components yield a different coordinatization for the same KS sets and therefore give different possible experimental implementations. For instance, 21–11 can be assigned vectors generated by the set of components {0, +3, ±*i*}, or {0, ±1, ±*i*}, or  any other from Fig. 2(c–h). In Appendix A of ref.^43^ we show how components of vectors of a given coordinatization of a 21–11 KS set directly generate quantum states via which we might implement the set.

In Fig. 2, the hypergraphs from the 636–1657 class are not so uniformly distributed as those from the 60–105 simply because a generation from such a big master requires orders of magnitude more time. Still, an especially sparse population of hypergraphs with 38 to 117 edges indicates an uneven probability of getting individual KS critical sets from the master set, similarly to the 300–675 probabilities (recall the gaps we obtained in^39,46^).

**Figure 3:**
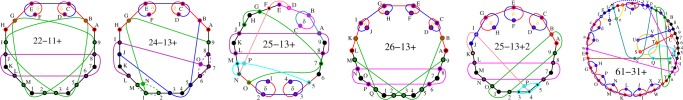
MMP hypergraphs of KS critical sets derived from the 636–1657 master that are not subgraphs of 60–105. The first four share the vertex components with the latter master while the last two do not; *δ*'s denote the *δ*-feature introduced in^39^.

Five smaller hypergraphs from the class 636–1657 that are not subgraphs of 60–105 are given in Fig. 3. What is interesting about them is that the first four of them, although obtained from the {0, ±1, ±*i*, ±2} based master set, also have a {0, ±1, ±*i*} coordinatization. For instance, **22**–**11** + 1234, 4567, 789A, ABCD, DCEF, FEGH, HIJK, KLM1, 68JL, 29GM, 35BI. {1 = {1, 1, 1, 1}, 2 = {1, 1, −1, −1}, 3 = {1, −1, i, −i}, 4 = {1, −1, −i, i}, 5 = {1, 1, i, i}, 6 = {1, i, 1, −i}, 7 = {−1, i, 1, i}, 8 = {1, i, −1, i}, 9 = {1, 0, 1, 0}, A = {0, 1, 0, −1}, B = {i, 0, 1, 0}, C = {-i, i, 1, i}, D = {1, 1, i, 1}, E = {i, −i, 1, i}, F = {i, i, 1, −i}, G = {0, 1, 0, 1}, H = {1, 0, i, 0}, I = {0, i, 0, 1}, J = {i, 1, 1, i}, K = {1, i, −i, −1}, L = {1, −i, i, −1}, M = {1, −1, −1, 1}} (‘+’ in **22–11** + means that it is a 2nd **22–11** critical in the class).

The fifth hypergraph does not have such a coordinatization: **25–13** + **2** 1234, 4567, 789A, ABCD, CDEF, EFGH, GHIJ, JKLM, MNO1, 29BO, 345P, 4PIK, 68LN . (‘+**2**’ in **22–13** +** 2** means that it is a 3rd **22–13** critical in the class).

The **61–31**+ critical, with more vertices than any KS set from the 60–105 subclass, possesses a {0, ±1, ±*i*, 2} coordinatization. **61–31**+ 1234, 5674, 89A7, BCD6, EFGH, IJKL, MNKL, OPMN, QR23, STUV, WXIJ, OPGH, UVDA, STC9, YZWX, abcd, eQRF, fgcd, hijk, lmkg, nojf, pome, qr51, stuZ, vurY, wabE, xtB8, ywvq, yxsp, zynl, zyhi.

The biggest critical with 31 edges from the 60–105 subclass has 58 vertices.

There are also KS hypergraphs with more than 60 vertices and with a {0, ±1, ±*i*} coordinatization, e.g., those obtained from the 156–249 master. The following 61–33 is one of the smallest of them. **61–33**+ 1234, 1256, 789A, 7BCD, 8BE5, F9GH, FAIJ, KLMN, KLOP, QROS, QTUV, WRXY, WTZ4, abTc, adIe, aTfg, achi, bdE6, jklm, jlni, XopN, Zq34, oYfr, kmhs, q4IJ, Gtuv, wxtHwyIe, xyCD, Mpns, zOrg, zOSP, uUVv.

The KS sets above have parity proofs. Actually, all KS criticals from 18–9 through 35–19 do have them, while none from 66–35 through 284–193 has one. In the region from 36–19 through 65–35 (*mixed parities*), KS criticals with odd number of edges might or might not have them (KS sets with even number of edges cannot have a parity proof by definition). Parities are indicated in Fig. 2.

Most of the hypergraphs from the 636–1657 class exhibit the *δ*-feature we recognized in Sec. V and Fig. 5 of ^39^ and indicated in Fig. 3–when pairs of edges share two vertices i.e. intersect each other twice at two vertices.

Global distribution of the hypergraphs shown in the inset (b) of Fig. 2 exhibits the agglomeration below the line which connects the smallest and largest criticals. Also, the number of vertices per edge decreases toward both smallest and biggest vertices. Both features are consistent with those of 300–675 and 148–265 classes shown in Fig. 1(b) and in Fig. 4(b) of the Supplementary Material.

## Extension to Higher Dimensions

**Figure 4:**
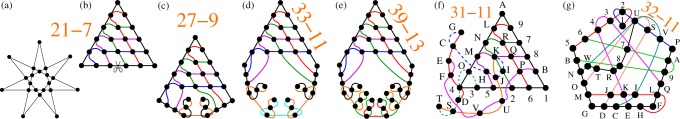
6D *ω* KS criticals sets; (**a**) a star representation of 21–7 from^12^; (**b**) its isomorphic triangular equivalent from^39^; (**c**,**d**) the only two other criticals in the *ω* class within the *ω*-coordinatization; pairs of *δ*-triples comes to the triangle at the scissor indicated point; (**e**) 39–13 generated within the *ω*^2^-coordinatization from the 591–1123 master set and is contained neither in the *ω* 216–153 nor in the *ω*^2^ 81–162 class; (**f**) the second smallest from the 81–162 class presented so as to show the “remnants” of the 21–7 triangle; (**g**) the third smallest set from the class shown in the standard maximal loop representation; MMP hypergraph encodings and their coordinatizations are given in the Supplementary Material.

An automated generation of KS sets of the kind presented in the previous section can be extended to any dimension. Here we give an example whose generation escaped all previous attempts via other methods. It is related to the star-like 6D 21–7 KS set^12^ which was implemented by^13^. They made use of *ω*^2^-coordinatization (where $\omega ={e}^{2\pi i/3}=(\,-\,1+i\sqrt{3})$/2) defined by the vector components from the set {0, 1, *ω*, *ω*^2^}. In^39^ it was shown that the set can be given a triangular hypergraph representation and a simpler *ω*-coordinatization based only on the components from the set Ω = {0, 1, *ω*}. All previous attempts^12,39^ to find a KS class, or a master set to which the 21–7 set might belong, failed. In^39^, a huge polytope based 6D class was generated, but the 21–7 provably did not belong to it. The vector generation based on the latter *ω*-coordinatization gives an *ω*-master set 216–153 and its class right away, though. The master set is connected, i.e., it consists of a single KS set and does not contain any unconnected non-KS sets as the master sets shown in Figs 1 and 2 do. It means that the *ω*-coordinatization exhaust all possible assignments from Ω. Still, the class 216–153, apparently contains only three critical KS sets, shown in Fig. 4(a–c), meaning that after several CPU years of generating criticals from the master set 216–153 we did not obtain any other critical–only a myriad of isomorphic replicas of those three. All three of them have parity proofs.

A natural question arises: is there at least the next critical in the series, i.e., the 39–13 one with an additional pair of *δ*-triples, with however small probability of appearing? (By the very definition of the MMP hypergraphs, 6D ones can have up to four vertices that share two edges). The answer is in the negative, because ascribing of the *ω*-coordinatization to its hypergraph fails. But we are able to vector generate 39–13 from the *ω*^2^-coordinatization–the (d)-heptagon from Fig. 4 is then extended to a nonagon with two additional *δ*-triples at the bottom as shown in Fig. 4(e). What geometrical property of the 216–153 master limits it to just three criticals 21–7, 27–9, and 33–11 and why the generation of the 39–13 critical requires a bigger *ω*^2^-coordinatization, remain open questions.

**Figure 5:**
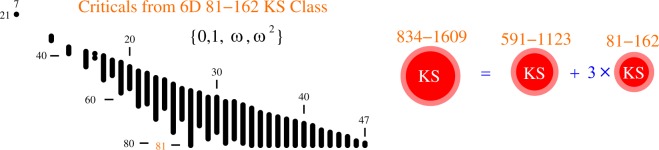
Distribution of 2.5 × 10^7^ non-isomorphic KS criticals from the 6D *ω*^2^ 834–1609 KS class. See the caption of Fig. 1 for the representation of the criticals via dots and lines in the main figure. Vertex units are scaled to 1/4 of edge units for a compact presentation. The 834–1609 master consists of two submasters–see text.

The *ω*^2^-coordinatization generates an 834–1609 master set. Unlike the *ω*-master set 216–153, the 834–1609 *ω*^2^-master set consists of four unconnected KS sets as shown in Fig. 5–one 591–1123 and three mutually isomorphic 81–162 sets, and thus amounts to just two sets. We have not yet generated the 591–1123 KS criticals since it requires too many CPU years on supercomputers due to their numerosity and dimensionality.

## Discussion

In this paper we designed an unprecedentedly fast and efficient way of generating Kochen-Specker (KS) sets, a kind of quantum contextual sets, for application and implementation in quantum communication and computation. The design is based on our discovery^43^ that fairly simple sets of vector components can generate, with a suitable algorithm, master KS sets within seconds on any PC. In this paper we provide methods of exhaustive generation of smaller critical KS subsets from such master sets. We call this kind of generation of both master KS sets and smaller KS sets they contain, the *vector generation of* KS *sets*.

We present it in detail for 4D Hilbert space, although the method is applicable to any dimension. As an example, we provide a generation of the star/triangle 6D class which the previous methods failed to provide. We also discuss a 3D case.

With vector generation, we effectively circumvent solving systems of nonlinear equations to find coordinatizations for generated KS hypergraphs that we faced in our previous approaches starting with^29^. In the latter approach, we first generated MMP hypergraphs and then searched for possible coordinatizations for them. The vector generation approach is its inverse–we first generate a coordinatization and then search for possible MMP hypergraphs for them. This reduces finding of KS masters together with their coordinatizations from CPU years of calculations on supercomputers to minutes on a PC.

At the present stage of our research, we combine two ways of finding sets of simple vectors for vector generation of KS sets: random computer searching, and taking over the coordinatization of master KS sets previously found via polytope symmetries or Pauli operator structures. In order to find an optimal approach to a successful vector generation of KS master set we have to find a balance between desired features of the KS classes we want to generate from a chosen master set and the required complexity of the their computer generation on supercomputers via our programs. For this purpose we extract many characteristic features of the sets while processing them.

We found that vector components exhaustively generate a coordinatization of a KS master in the sense of employment of *all* possible vectors. This, in most cases, results in a master set split into several unconnected sets as shown in Figs 1, 2 and 4. When a master splits into two unequally big KS submasters as in the 6D example given in Fig. 4 this enables us to generate smaller criticals because the original master 834–1609 and/or the bigger submaster 591–1123 are computationally unfeasible on a supercomputer. In 4 dimensions, vector generation of criticals from the 972–1852 KS master set, i.e., the 636–1657 submaster, is perfectly feasible as shown in Fig. 2. Also the classes 300–675 and 148–265 (see the Supplementary Material), even when enlarged in volume and scope by the vector generation of master sets which contain them, stay computationally feasible.

We also obtained and relied on a number of generic features of KS sets.

Complex components of vertices of the 4D KS classes are in the majority of cases linked to their implementation by means of two qubits, on the one hand, and to their *δ*-feature, on the other, where the latter feature means that pairs of edges share two vertices, i.e., intersect each other twice. Exceptions are among others, the smallest KS sets which can acquire both complex and real coordinatization as, e.g., the 18–9 KS set^28^. Real components of vertices mostly lead to spin-$\frac{3}{2}$ particle implementation, in the sense that corresponding operators are not qubit-describing ${ {\mathcal H} }^{2}\otimes { {\mathcal H} }^{2}$ Pauli operators.

The *δ*-feature stems directly from the *n* − 2 hypergraph condition within the definition of MMP hypergraphs given in MMP Hypergraph Language. This implies that edges of 3D MMP hypergraphs can intersect each other in at most 3 − 2 = 1 vertex. In addition, in^29^, we proved that the size of minimal loops of adjacent edges is not 2 as in 4 or 6D (exhibiting the *δ*-feature as in Fig. 3), but 5 (pentagon). These two features make 3D MMP hypergraphs computationally different from, e.g., 4D ones. Thus, although we can obtain 3D master hypergraphs as we pointed out above, we first have to develop new algorithms that would enable us to generate 3D criticals from them. This is a work in progress, which is the reason why we have not included any 3D results in this paper.

As a general rule, also for higher dimensions^39^, bigger KS sets do not have parity proofs at all, while the very small ones with odd number of edges all have them, as shown in Figs 1 and 2. Among the criticals of the 148–265 class, which does not have really small sets, not a single set with a parity proof was generated.

Sets generated from small master sets, like 4D 60–74, 60–105, 73-? and 84-?, and 6D 81–162 exhibit a flat cutoff as shown in Figs 1(a), 2(b) and 5, and Fig. 2 of the Supplemental Material, and form a saw-toothed distribution as shown in Fig. 2(b) of the Supplemental Material; this feature enabled us to fill in the gap between the upper and lower clusters of the 300–675 class.

All classes generated from sufficiently big master sets, exhibit a spindle-like distribution of sets with an agglomeration of criticals below the line that connects the smallest and largest of them as shown in Figs 1(a), 2(a) and 5.

The 96–96 KS set is found to properly contain all sets from the 60–74 (60–75) class. The big gap between 82 (41) and 211 (127) vertices (edges) in the 300–675 KS class we obtained in^39,46^ is filled in as shown in Fig. 1. The 96–96 KS sets form a subclass of the 300–675 KS class. Missing KS sets from the 147–265 KS class in^39,46^ are obtained.

In 6D the *ω*-coordinatization of the star/triangle 21–7 KS set^12,39^ is found to generate a master set 216–153 which contains only three criticals 21–7, 27–9, and 33–11 shown in Fig. 4(a–d). The *ω*^2^-coordinatization generates two KS sub-classes 591–1123 and 81–162; the distribution of the latter is shown in Fig. 5. The former is too big to be generated within the scope of this paper, but we confirmed that the 39–13 critical shown in Fig. 4(e) belongs to the former and not to the latter class.

In the end, we would like to recall that just over 50 years have passed since Simon Kochen and Ernst P. Specker formulated their KS theorem^45^ and that for 36 years thereupon only three 4D KS sets were found. The results obtained since then and partly in this paper prove the power of computer science in and interdisciplinary approaches to the field.

## Methods

The MMP hypergraph language, and that algorithms and programs developed for it, are our general method for processing and categorising KS sets and other properties represented by hypergraphs. By using exactly one line of text for each MMP hypergraph, we manipulate a collection of millions of MMPs with standard text processing tools as well as distribute pieces of the collection to different CPUs for massive parallel processing. Most of our programs work with arbitrary hypergraphs and can be useful for any project using hypergraphs for knowledge representation. While we encourage the use of MMP notation because it is well-defined and convenient, we can also translate to and from other hypergraph representations.

All our programs are freely available from our repository^47^, and with them a researcher can reproduce the results reported in this paper and its Supplemental Material. Any of our programs can process an arbitrary number of lines each consisting of ASCII characters that represent KS sets in the form of KS hypergraphs. There are no inherent limitations in the number of vertices (vectors) or edges in which vertices are organized (mutually orthogonal *n*-tuples of vectors determining the dimension *n* of the Hilbert space in which vectors reside).

Our programs have many different options available, which are documented via the –help for each of them. Their most important functions are as follows. Program Vecfind generates master sets from the input vector components, like {−1, 0, 1}, or verifies whether given KS hypergraphs can be assigned a coordinatization based on such sets. Mmpstrip strips a specified number of edges from a given KS sets or adds them. Shortd reduces collections of KS sets to non-isomorphic ones, thus eliminating duplicates. Mmpsubgraph verifies whether a given hypergraph is a subgraph of another bigger one. States01 filters out KS sets and critical KS sets from a given collection of sets. Loop generates the biggest loop for a given KS set which enables us to draw its figure by a program written in Asymptote or manually in Xfig. Note that one can easily read the ASCII string KS hypergraph representation off any figure even when no ASCII characters are assigned to vertices in the figure. What characterises a KS hypergraph is its structure, not a specification of characters or coordinates assigned to vertices. All these programs are written in C, developed in^26,29,31,32,46,48–50^, and extended here. We also used parity-proof algorithms and programs developed in^23,30,33,37^. Programs allow standard inputs and outputs and can be piped into each other.

## Supplementary information

Supplementary Info File #1

## Data Availability

Our programs are freely available at our repository http://goo.gl/xbx8U2. The datasets generated during the current study are available from the corresponding author on reasonable request.
