# Supplementary material for: Automated generation of Kochen-Specker sets
Source: Sci Rep. 2019 May 1;9:6765. doi: 10.1038/s41598-019-43009-9 (PMC6494998; doi:10.1038/s41598-019-43009-9)
Supplement: Supplementary file 1 — Supplementary Info File #1 [file 41598_2019_43009_MOESM1_ESM.pdf]

Supplementary Material to
Automated generation of Kochen-Specker sets

Mladen Pavičić[1,2,*], Mordecai Waegell[3], Norman D. Megill[4], and P.K. Aravind[5]

[1]Department of Physics—Nanooptics, Faculty of Math. and Natural Sci. I, Humboldt University of Berlin, Germany
[2]Center of Excellence CEMS, Photonics and Quantum Optics Unit, Ruđer Bošković Institute, Zagreb, Croatia.
[3]Institute for Quantum Studies, Chapman University, Orange, CA 92866, U.S.A.
[4]Boston Information Group, Lexington, MA 02420, U.S.A.
[5]Physics Department, Worcester Polytechnic Institute, Worcester, MA 01609, U.S.A.
[*]mpavicic@physik.hu-berlin.de

{-1,0,1}-Coordinatization: 40-32 KS Class

Program VECFIND with option `master` gives the following encoding of the **40-32** master hypergraph: `v40e32` 1234, 1256, 1378, 19A4, 23BC, 2DE4, FG34, FG56, F5HI, FJK6, G5LM, GNO6, DE78, D7OI, D8JL, E7KM, E8NH, 9ABC, 9BOH, 9CKL, PQRS, PTUV, WXRY, WZaV, ABJM, ACNI, bXUc, bZdS, eQac, eTdY, NOHI, JKLM.{1={0, 0, 0, 1}, 2={0, 0, 1, 0}, F={0, 0, 1, 1}, G={0, 0, 1, -1}, 3={0, 1, 0, 0}, D={0, 1, 0, 1}, E={0, 1, 0, -1}, 9={0, 1, 1, 0}, P={0, 1, 1, 1}, W={0, 1, 1, -1}, A={0, 1, -1, 0}, b={0, 1, -1, 1}, e={0, -1, 1, 1}, 4={1, 0, 0, 0}, B={1, 0, 0, 1}, C={1, 0, 0, -1}, 7={1, 0, 1, 0}, X={1, 0, 1, 1}, Q={1, 0, 1, -1}, 8={1, 0, -1, 0}, T={1, 0, -1, 1}, Z={-1, 0, 1, 1}, 5={1, 1, 0, 0}, a={1, 1, 0, 1}, U={1, 1, 0, -1}, d={1, 1, 1, 0}, N={1, 1, 1, 1}, J={1, 1, 1, -1}, R={1, 1, -1, 0}, K={1, 1, -1, 1}, O={1, 1, -1, -1}, 6={1, -1, 0, 0}, S={1, -1, 0, 1}, Y={-1, 1, 0, 1}, V={1, -1, 1, 0}, L={1, -1, 1, 1}, H={1, -1, 1, -1}, c={-1, 1, 1, 0}, I={1, -1, -1, 1}, M={-1, 1, 1, 1}}.

Its graphical representation is given in Fig. 1

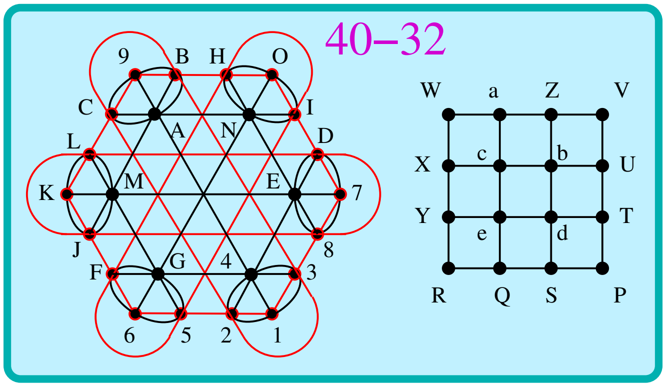

Figure 1. Graphical representation of the 40-32 master set with {-1,0,1}-coordinatization. It consists of two disconnected hypergraphs: the left one is isomorphic to Peres' 24-24 KS hypergraph ([1–3]) and the right one is a non-KS hypergraph which is generated to exhaust {-1,0,1}-coordinatization. The smallest 4D KS set 18-9, first found in[4], is indicated in red. For the other five critical KS sets see[3].

Program MMPSTRIP generates 3,712 smaller KS sets which together with their 40-32 master set form the 40-32 KS class. Of those, 1,233 connected ones (up to 24 vertices and 24 edges) are isomorphic with the ones previously obtained from Peres' 24-24 KS set in[2].

Polytope Generation

600-Cell Generates the 60-74 KS Master Set

The 600-cell has 25 different 24-cells inscribed in it, in the way that a regular dodecahedron has five cubes inscribed in it. The inscribed 24-cells can be seen most easily in the basis table of the 600-cell, where each 24-cell can be seen as one of the blocks of 12 vectors (each representing an antipodal pair of vertices of this 24-cell). The vertices of the 600-cell can be partitioned into those of five disjoint 24-cells in ten different ways, with the rows and columns of Table 2 of[5] showing the different partitions. It

is important to note that although the 600-cell has many 24-cells in it, no 24-cell is accompanied by its dual. Thus, the 24-24 system described above is not a subset of the 60-75 master set.

120-Cell Generates the 300-675 KS Mater Set

The 120-cell {5,3,3} has 120 dodecahedra for its outer boundary, with three around each edge of the polytope. Alogether it has 120 pentagons, 1200 edges, and 600 vertices. These vertices, lying on a 3-sphere in 4D, come in antipodal pairs, and so give rise to just 300 distinct vectors or quantum states. These 300 vectors form 675 bases of four mutually orthogonal states, from which we obtain the 300-675 KS master set according to the prescription given in[6].

Polytope Generation of the 300-675 and 96-96 KS Classes and Their Features

In[6] we obtained a 300-675 KS master set from the 120-cell polytope. However, the set was too big for our parity proof program, so that we made use of a 96-96 subset to generate critical (smallest) KS sets [6, Table 5]. It is not too big for our MMPSTRIP, STATES01 and MMPSUBGRAPH programs[2,7], though, and we obtained all the results in this section by making use of these three programs. The MMP hypergraph string of the master set 300-675 itself is however too long to be reproduced here and the reader can retrieve it from our repository in[8].

Subsets of the master set 300-675 form the 300-675 KS class and in Fig. 1 in the main body of our paper, we give all the critical KS sets we obtained from this class.

The very construction of the 300-675 master set (presented in[6]) involved the 60-75 master set and therefore it constructively proved that the 300-675 KS class properly contains the 60-74 class. Namely, by stripping one edge at the time from the 60-75 master set we obtain seventy five 60-74 KS subsets. They turn out to be isomorphic to each other and actually merge into a single 60-74 set. Thus, both sets can be taken as master sets for smaller KS sets which form a class of their subsets and in the literature we called them 60-75 and 60-74 classes, respectively. They are identical, short of the very 60-75 set from the 60-75 class. This is a general feature of any master set in which all edges are symmetrically identical.

In[6] we called the 60-75 master set (first discovered in[9] and further elaborated in[5,10]), the 600-cell. In that paper we mostly considered the 96-96 set ("one of the reduced sets," actually the last one from Table 4 of the paper, i.e., $36_2 48_5 12_6 - 96_4$). It turns out that the 96-96 set properly contains the 60-75 set and therefore also the 60-74 set as well as all the sets from the 74 class. To see this, let us have a look at Table 1 of[6]. In the table, the 60 rays of the 60-75 are labelled E′ (they form a subset of the 300-675). We can construct the 96-96 so as to cancel 12-ray-blocks and from Table 4 we then see that E′ remains intact.

Another way to prove that the 96-96 subset contains the 60-75 master set and all KS criticals from the 60-74 class in addition to criticals not contained in the 60-74 class is to make use of our universal programs MMPSTRIP and STATES01. In this approach we obtain a number of results which are presented below and partly displayed in Fig. 2.

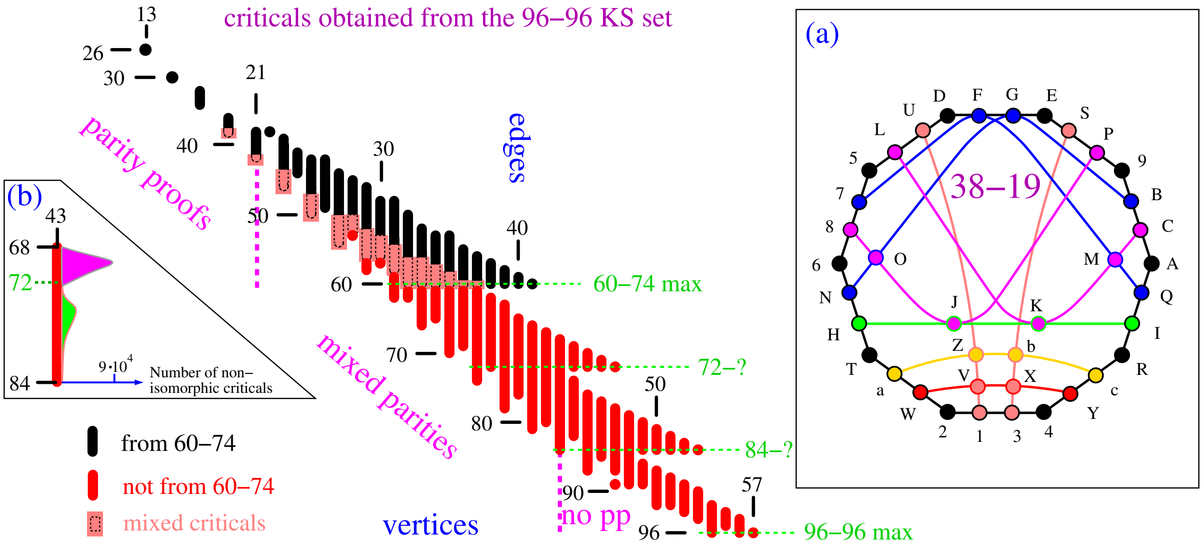

Figure 2. Distribution of 5×10^9 KS criticals obtained from the 96-96 KS subset of the 300-675 KS master set[6] via MMPSTRIP and STATES01 programs. For an explanation of differently colored criticals see text. Vertex units are scaled to 1/2 of edge units to give a more compact graphical presentation. "Mixed criticals" mean that only a portion of KS criticals with the shown vertices and edges are from the 60-74 class. Inset (a): the smallest critical hypergraph which is not a subgraph of the 60-74 master KS set. Inset (b): the distribution of all criticals with 43 edges. "no pp" means "no parity proofs."

The black criticals are from the 60-74 class. Squared pink rectangles with dashed lines mean that some of the criticals are from the 60-74 while the others are not. Criticals in red do not belong to the 60-74 class. We obtained all the criticals via the universal MMPSTRIP, STATES01, and MMPSUBGRAPH programs but we discerned 60-74 from non-60-74 criticals with an odd number of edges mostly via our parity proof program which is much faster than MMPSUBGRAPH. Criticals with an even number of edges which cannot be discerned by means of parity proof algorithms we discerned via STATES01. Criticals from the 60-74 cannot have more than 60 vertices which we visualized by means of the "60-74 max" dashed line. The 300-675 class contains the 60-74 class but it does not exhibit a cutoff shape. Its MMP hypergraphs contain edges with fewer and fewer vertices toward the opposite ends of their distribution. Also they exhibit agglomeration of criticals below the line which connects the smallest and largest hypergraphs. As we can see in Fig. 4(b), the 148-265 class possesses the same features.

The above cutoff shape of the 60-74 class appears to be characteristic of any subclass of a bigger class, which we tentatively call a *complete class* generated from a *complete master set*. Under *complete sets* we understand the ones obtained from an exhaustive usage of vector components to build their coordinatization. Then their distribution exhibits a spindle-shaped agglomeration elaborated on in Subsection **Vector Generation of KS Sets from Basic Vector Components** of the paper. E.g., in Fig. 2, from the distribution of the 96-96 criticals, in particular from its saw-toothed shape, we can, apart from the subclass 60-74, recognize two new subclasses 72-? and 84-? where "?" stands for the edges of the sub-master sets that we do not know as of yet. Also, through the cutoff at the 96 vertex level, we can see that the 96-96 class itself is a subclass of a bigger complete set, e.g., the 300-675 one. Our claim that the saw-toothed shapes are genuine effects and not consequences of insufficiently intensive generation of sets are supported by, first, the fact that among the last 15% of generated sets there were no new types belonging to the teeth area (only two from bottom left area), and, second, by two clearly pronounced sub-distributions of vertices related to the same edges and the base of each tooth. In Fig. 2, an example of such a double sub-distribution is shown in the Inset (b) for the edge 43. There is only one KS **73-43** critical (enneadecagon in the lower runs of the program LOOP): 6587,7wxr,rq1n,nlmk,koec,cb4P,PKGC,C9BA,AIEU,U#s3,3∗"g,gaYW,WVOT,TRSQ,Q/$z,zy!f, f(%d,d&h′,′u26,,,1234,DEFG,HIJK,LMNO,JFB8,XYSN,ZaRM,de73,fgc6,hi54,jHD9,iZXV,opL2, stpn,uvie,"!rb,#vmb,$%t4,)$l6,∗(o5,-/q5,#zxh,-)db,/&sc.

Another example of the distribution shown in the Inset (b) of Fig. 2, is of sets with 37 edges with an order of magnitude bigger sub-distribution of vertices than for the above 43 edges.

MMP hypergraph representations of the smallest KS criticals with an odd number of edges that do not belong to the 60-74 class, obtained from the 96-96 sets with parity proof programs, in particular two 38-19, 42-21, and 48-25, are given in [3, Fig. 8]. In Fig. 2, in the inset (a), the hypergraph figure of 38-19 is given. Its code reads: **38-19**: 2134,4cYR,RQIA,ABC9,9SPE, EFGD,DUL5,5786,6NHT,TaW2,,,HIJK,LMKC,NOGB,POJ8,QMF7,VWXY,Zabc,bXS3,ZVU1.

In Fig. 3 we give some MMP hypergraph representations of the smallest KS criticals with an even number of edges, which cannot be obtained by means of parity proof algorithms.

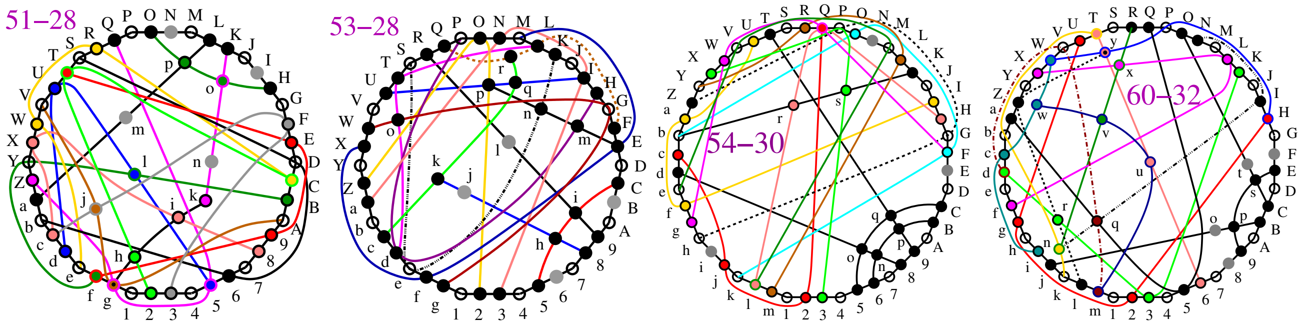

Figure 3. Four small KS criticals with an even number of edges. In contrast to small criticals with an odd number of edges they all have several vertices that belong to only one edge (denoted as grey dots).

They lack the symmetry many of KS criticals with an odd number of edges exhibit (Cf. [3, Figs. 3 and 8]). Their MMP hypergraphs run as follows.

51-28: 1234,4567,789A,ABCD,DEFG,GHIJ,JKLM,MNOP,PQRS,STUV,VWXY,YZab,bcde,efg1 ,,,2hTC,3Fjc,5lUd,5QZg,6bDS,8iXc,9fET,AgjV,BlYf,CRWe,HopO,Konk,Lpma,ghik.

53-28: ,,,aNI3,XMfE,5hiC,8hjk,9ilR,Emnp,gGoW,SenK,PdmH,UpqI,ckqr,QrLF,doTL,ZOp2.

54-30: ,,,2Qjc,3sPX,5oqC,6npB,8nod,9pqT,FQWg,FkOb,GhPZ,HQrl,IfaU,Ksrb,LmRY,MlVe.

60-32: ,,,2HUg,3Krd,5Qqa,6uyT,9psE,Bpoj,CstO,HPyW,INqn,LxXf,Rxvn,Tbnk,Vdqm,Wwci,Zrly,wvum.

The main loops are obvious from the figures (alphabetical order), and except for the first set we omit them. The loops are 14-gons for the first two and 16-gons for the last two sets. Some 58-32 and 59-32 have 17-gons as maximal loops but these are actually small as compared to many KS criticals from the upper part of the class shown in Fig. 1 of the paper whose maximal loops are 55-gons and bigger.

In[3,11] a uniform random generation of criticals with MMPSTRIP and STATES01 generated a cluster of sets with edges between 127 and 188 and vertices between 211 and 283. The programs did not generate a single critical in between this cluster and the 96-96 cluster. When we tried to find out the cause of the gap we first realised that none of the criticals from the upper cluster had a parity proof, so, we could not obtain them with a parity proof program. Then, we realised that a probability of finding criticals between the aforementioned criticals via a uniform random procedure in the 300-675 class is extremely low (unlike in any other class in any dimension[3]) and that a random generation of each of them might take up to 3 months. However, following the above 96-96 saw-toothed feature, we conjectured that an analogous feature repeats throughout the 300-675 class up to the upper cluster.

We verified the conjecture in the following manner. We first let 100 jobs run in parallel for 3 months and obtained a number of criticals in between the former upper and lower clusters. Then we traced the subsets they were generated from. When a considerable number of criticals pointed to particular subsets, we ran up to 200 STATES01 jobs on these subsets in parallel. This proved fruitful and we filled in the gap, denoted by question marks in Fig. 9[3] and Fig. 2 of[11], and obtained evenly distributed critical KS set shown in Fig. 1 of the paper.

We found no parity proof among criticals bigger than 82-41, so the upper part of the 300-675 KS class is invisible to the parity proof algorithms.

The biggest maximal loop we found is a 57-gon for a 283-188 critical.

The 300-675 KS class has a real coordinatization, i.e., vectors corresponding to vertices of their MMP hypergraphs are from $\mathbb{R}^4$. The consequence is the structural simplicity of the hypergraphs. In particular, they all lack the δ-feature of two edges sharing two vertices characteristic of the hypergraphs from the 60-105 class that has the complex coordinatization.

Witting Polytope Generates the 148-265 KS Class

Complex coordinatization does not automatically furnish the hypergraphs with intricate features, as the 148-265 class shows[3,11]. The 148-265 KS class sits in complex 4D Hilbert space, and the two classes 300-765 and 148-265 are completely disjoint, making the latter class particularly interesting for designing experiments with qubits residing in complex Hilbert space. The class is obtained from a polytope with coordinatization in $\mathbb{C}^4$, called the *Witting polytope*.

The well-known Penrose dodecahedron 40-40 KS set in 4D complex Hilbert space is related to the Witting Polytope, through the Majorana representation of spin states to the regular dodecahedron in 3-dimensional Euclidean space, and through a particular projection, to the root structure of the Lie algebra E8 in the 8-dimensional Euclidean space[12]. Its coordinatization can be obtained from the set $\{0, \pm 1, \pm\omega, \pm\omega^2\}$, where $\omega = e^{2\pi i/3}$.

Penrose's 40-40 set is not critical, and we exhaustively generated all KS subsets it contains. The outcome turns out to be of importance for the definition of critical sets since all criticals contain all 40 vertices of the master set and are obtained by removing only edges. This shows that the vertex criticality criterion "A KS set is critical if by deleting any of its vertex it turns into a non-KS set" which can be found in the literature (for example[13,14]) is too restrictive.

On the other hand, it is important because it is an extreme example of a flat cutoff feature with no vertical (vertex) distribution of hypergraphs.

None of the 40-40 subsets has a parity proof, so, we made use of the hypergraph program STATES01 to generate them. The 40-40 master set from [12, Table 3] is represented by the following MMP hypergraph 1BLV,bDO7,b4UJ,bGAR,1468, BDKF,BGEI,LUNP,LOQS,1A35,D3MZ,4EMc,UK2e,GQ2a,ANCX,O6CY,6KdT,ENd9,Q3dH,M2CW,bVdW, BJCH,UI3Y,GP6Z,LRMT,1729,4FQX,EeO5,NaD8,ASKc,VaYc,VXZe,J5aT,JS9Z,I7XT,I8SW,P5FW, P7cH,R8eH,RF9Y.

Via exhaustive stripping by means of the program MMPSTRIP and filtering through the program STATES01, we obtain all subgraphs and all critical subgraphs among them, as shown in Table 1.

Table 1. All subgraphs, and all critical subgraphs among them, generated from the Penrose 40-40 dodecahedron hypergraph.

40-40	all dodecahedron subgraphs (all have 40 vertices)																	criticals		
edges	40	39	38	37	36	35	34	33	32	31	30	29	28	27	26	25	24	23	25	24	23
No. of sets	1	1	2	5	15	47	160	553	1870	5822	16208	39593	82944	144315	193818	164536	24948	56	60752	24265	56

In Fig. 4 we give a figure of one of the 56 smallest 40-23 KS criticals. Its MMP representation runs as follows (maximal loop symbols can be read off from Fig. 4): ,,, 89A3,EFG4,HIJ2,KLM1,QRSJ,WOH6,XYZ8,abc5,cVPD,cZQ1,bYN2, dWU1,eVT2. All 40-40 criticals have decagons as their maximal loops.

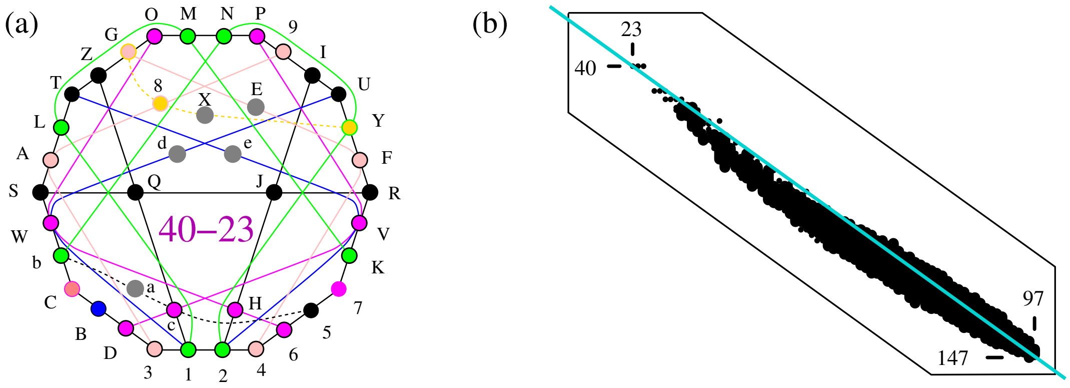

Figure 4. (a) 40-23 critical; ASCII symbols of vertices are taken directly from the computer generated MMP hypergraph. Grey dots depict vertices that belong to just one edge; (b) Distribution of 250140 KS criticals from the 148-265 class which shows the same kind of agglomeration of criticals below the line which connects the smallest and largest of them as in the 300-675 class in Fig. 1 of the paper. Also, again as in the 300-675 class, the number of vertices per edge decreases toward the opposite ends of the distribution.

The 148-265 KS master set was in[8] constructed with coordinates from $\{0, \pm i, \pm 1, \pm\omega, \pm\omega^2, \pm i\omega^{1/\sqrt{3}}, \pm i\omega^{2/\sqrt{3}}\}$ [12, Table 2]. However, in[3], we showed that the set $\{0, \pm 1, \pm\omega, \pm\omega^2\}$ suffices.

As shown in Fig. 4(b), the criticals we obtained in the 148-256 class and which do not belong to the 40-40 ones have vertices in the span from 49 to 147 and edges from 27 to 97 and exhibit spindle-shaped agglomeration similar to the 300-675 class. The sets with 49 vertices might belong to a subclass similar to the 40-40 one, but we have not explored them further.

The maximal loops of the criticals go up to 36-gons, and their hypergraphs are therefore mostly too big to be efficiently visualized. One of the smallest non-40-40 ones, a 49-27 critical, has been presented in [3, Fig. 10] and [11, Fig. 2].

No set we generated from either the 300-675 class or the 148-265 class contains edges that share more than one vertex. Some sets from other classes do contain such edges, which we call the δ-feature, and it proves to be characteristic of the 60-105 class in particular.

6D MMP Hypergraph Encoding

6D MMP hypergraph encoding of the critical KS set **21-7** with ω-coordinatization is given in[15] and its ω^2-coordinatization in[16]

MMP encoding of the next two KS criticals (Fig. 4(c,d) of the paper) are:

27-9 123456, 1789AB, CDEFGH, CIJKAL, DMI9NO, MF7PKQ, E234GH, R5J8OQ, RPNLB6. {1={0, 0, 0, 0, 0, 1}, 2={1, ω, 1, 0, 0, 0}, 3={ω, 1, 1, 0, 0, 0}, 4={1, 1, ω, 0, 0, 0}, 5={0, 0, 0, 0, 1, 0}, 6={0, 0, 0, 1, 0, 0}, 7={0, ω, ω, 1, 1, 0}, 8={1, 1, ω, ω, 0, 0}, 9={ω, 0, 1, ω, 1, 0}, A={ω, 1, 0, 1, ω, 0}, B={1, ω, 1, 0, ω, 0}, C={0, 0, 1, 0, 0, 0}, D={0, 1, 0, 0, 0, 0}, E={0, 0, 0, 1, 1, ω}, F={1, 0, 0, 0, 0, 0}, G={0, 0, 0, 1, ω, 1}, H={0, 0, 0, ω, 1, 1}, I={1, 0, 0, ω, ω, 1}, J={1, ω, 0, 1, 0, ω}, K={0, 1, 0, ω, 1, ω}, L={ω, ω, 0, 0, 1, 1}, M={0, 0, 1, 1, ω, ω}, N={1, 0, ω, 0, 1, ω}, O={ω, 0, ω, 1, 0, 1}, P={0, 1, ω, 0, ω, 1}, Q={0, ω, 1, ω, 0, 1}, R={ω, 1, 1, 0, 0, ω}}

33-11 123456, 789ABC, DEFGHC, IJKLHB, MNOLGA, PQRMNO, STUKF9, VWUJE8, XWTID7, PQR456, XVS123. {1={1, 0, 0, 0, 1, ω}, 2={1, 0, 0, 0, ω, 1}, 3={ω, 0, 0, 0, 1, 1}, 4={0, 1, ω, 1, 0, 0}, 5={0, 1, 1, ω, 0, 0}, 6={0, ω, 1, 1, 0, 0}, 7={0, 0, 1, 1, ω, ω}, 8={0, 1, 0, ω, 1, ω}, 9={0, 1, ω, 0, ω, 1}, A={1, 0, 0, 0, 0, 0}, B={0, ω, 1, ω, 0, 1}, C={0, ω, ω, 1, 1, 0}, D={ω, 0, 1, ω, 1, 0}, E={ω, 1, 0, 1, ω, 0}, F={1, ω, 1, 0, ω, 0}, G={0, 0, 0, 0, 0, 1}, H={1, 1, ω, ω, 0, 0}, I={ω, 0, ω, 1, 0, 1}, J={1, ω, 0, 1, 0, ω}, K={ω, 1, 1, 0, 0, ω}, L={0, 0, 0, 0, 1, 0}, M={0, 1, ω, ω, 0, 0}, N={0, ω, 1, ω, 0, 0}, O={0, ω, ω, 1, 0, 0}, P={1, 0, 0, 0, ω, ω}, Q={ω, 0, 0, 0, 1, ω}, R={ω, 0, 0, 0, ω, 1}, S={0, 0, 0, 1, 0, 0}, T={1, 0, ω, 0, 1, ω}, U={ω, ω, 0, 0, 1, 1}, V={0, 0, 1, 0, 0, 0}, W={1, 0, 0, ω, ω, 1}, X={0, 1, 0, 0, 0, 0}}

Of MMP encoding with ω^2-coordinatization we just give

31-11 123456, 1789AB, CDEFGH, CIJ96K, L2M4NO, L2EPQH, R7MFGO, R7SPQB, 2T8JNU, 7TSV5K, DI3VAU. {1={ω, 1, 1, ω^2, ω, 1}, C={ω, 1, 1, ω, 1, ω^2}, L={ω, 1, 1, ω, ω^2, 1}, R={1, ω^2, ω^2, 1, 1, 1}, 2={ω, 1, ω^2, 1, 1, ω}, 7={ω^2, ω, 1, ω, 1, 1}, D={ω, 1, ω^2, 1, ω, 1}, I={1, ω^2, 1, ω, ω, 1}, T={1, ω^2, 1, 1, 1, ω^2}, 3={ω^2, 1, ω, ω, 1, 1}, M={ω^2, 1, ω, 1, ω, 1}, S={ω^2, 1, ω, 1, 1, ω}, E={1, ω, ω^2, ω, 1, 1}, 8={1, ω, ω^2, 1, ω, 1}, J={ω^2, 1, 1, 1, ω^2, 1}, V={1, ω, ω, 1, ω^2, 1},

9=\{ω, ω^2, ω, 1, 1, 1\}, P=\{1, ω, 1, 1, ω^2, ω\}, F=\{1, ω, 1, 1, ω, ω^2\}, A=\{1, 1, 1, 1, 1, ω\}, 4=\{1, ω, 1, 1, 1, 1\}, 5=\{1, 1, 1, ω, ω^2, ω\}, Q=\{1, 1, 1, ω^2, 1, ω^2\}, N=\{1, 1, 1, ω^2, ω, ω\}, G=\{1, 1, 1, ω^2, ω^2, 1\}, 6=\{1, 1, ω, 1, ω, ω^2\}, O=\{1, 1, ω, ω, 1, ω^2\}, H=\{ω^2, ω^2, 1, 1, 1, 1\}, B=\{1, 1, ω, ω, ω^2, 1\}, K=\{1, 1, ω^2, ω^2, 1, 1\}, U=\{ω, ω, 1, ω^2, 1, 1\}\}

References

1. Peres, A. Two simple proofs of the Bell-Kochen-Specker theorem. *J. Phys. A* **24**, L175–L178 (1991).
2. Pavičić, M., Megill, N. D. & Merlet, J.-P. New Kochen-Specker sets in four dimensions. *Phys. Lett. A* **374**, 2122–2128 (2010).
3. Pavičić, M. Arbitrarily exhaustive hypergraph generation of 4-, 6-, 8-, 16-, and 32-dimensional quantum contextual sets. *Phys. Rev. A* **95**, 062121–1–25 (2017).
4. Cabello, A., Estebaranz, J. M. & García-Alcaine, G. Bell-Kochen-Specker theorem: A proof with 18 vectors. *Phys. Lett. A* **212**, 183–187 (1996).
5. Waegell, M., Aravind, P. K., Megill, N. D. & Pavičić, M. Parity proofs of the Bell-Kochen-Specker theorem based on the 600-cell. *Found. Phys.* **41**, 883–904 (2011).
6. Waegell, M. & Aravind, P. K. Parity proofs of the Kochen-Specker theorem based on 120-cell. *Found. Phys.* **44**, 1085–1095 (2014).
7. Pavičić, M., Merlet, J.-P., McKay, B. D. & Megill, N. D. Kochen-Specker vectors. *J. Phys. A* **38**, 1577–1592 and 3709 (corrigendum) (2005).
8. Megill, N. D. & Pavičić, M. Master sets and programs repository (2017). Http://goo.gl/xbx8U2.
9. Waegell, M. & Aravind, P. K. Critical noncolorings of the 600-cell proving the Bell-Kochen-Specker theorem. *J. Phys. A* **43**, 105304–1–13 (2010).
10. Megill, N. D., Fresl, K., Waegell, M., Aravind, P. K. & Pavičić, M. Probabilistic generation of quantum contextual sets. *Phys. Lett. A* **375**, 3419–3424 (2011).
11. Megill, N. D. & Pavičić, M. New classes of kochen-specker contextual sets (invited talk). In *Proceedings of the 40th* MIPRO *Convetion held on 22-26 May 2017*, IEEE Xplore Digital Library, 195–200 (IEEE, 2017). ISBN: 9781509049691.
12. Waegell, M. & Aravind, P. K. The Penrose dodecahedron and the Witting polytope are identical in $\mathbb{CP}^3$. *Phys. Lett. A* **381**, 1853–1857 (2017).
13. Ruuge, A. E. New examples of Kochen–Specker-type configurations on three qubits. *J. Phys. A* **45**, 465304–1–17 (2012).
14. Zimba, J. & Penrose, R. On Bell non-locality without probabilities: More curious geometry. *Stud. Hist. Phil. Sci.* **24**, 697–720 (1993).
15. Pavičić, M. Nondestructive interaction-free atom-photon controlled-NOT gate. *Phys. Rev. A* **75**, 032342–1–8 (2007).
16. Lisoněk, P., Badziąg, P., Portillo, J. R. & Cabello, A. Kochen-Specker set with seven contexts. *Phys. Rev. A* **89**, 042101–1–7 (2014).
